# Genetic and Epigenetic Features of Uveal Melanoma—An Overview and Clinical Implications

**DOI:** 10.3390/ijms241612807

**Published:** 2023-08-15

**Authors:** Daria Pašalić, Tamara Nikuševa-Martić, Ankica Sekovanić, Snježana Kaštelan

**Affiliations:** 1 Department of Medical Chemistry, Biochemistry and Clinical Chemistry, School of Medicine, University of Zagreb, 10000 Zagreb, Croatia; daria.pasalic@mef.hr; 2 Department of Biology and Genetics, School of Medicine, University of Zagreb, 10000 Zagreb, Croatia; 3 Institute for Medical Research and Occupational Health, 10000 Zagreb, Croatia; asekovanic@imi.hr; 4 Department of Ophthalmology and Optometry, School of Medicine, University of Zagreb, 10000 Zagreb, Croatia; snjezanakastelan@yahoo.com; 5 Department of Ophthalmology, Clinical Hospital Dubrava, 10000 Zagreb, Croatia

**Keywords:** clinical implications, genetics, liver function tests, miRNA, protein biomarkers, uveal melanoma

## Abstract

Uveal melanoma (UM) is rare, but it is the most common primary intraocular malignancy among adults. This review represents the molecular, genetic, and immunobiological mechanisms involved in UM carcinogenesis and progression, as well as data about the association of chromosomal changes, genetic mutations, selective proteins, and biochemical biomarkers with the clinical implications of UM. Genetic analysis has the potential to identify patients with a high risk of UM metastasis, enabling management that is more effective and allowing for the follow-up of patients. Advancements in molecular characterization of UM offer opportunities to develop targeted therapeutic strategies by focusing on relevant signaling pathways. Changes in miRNA expression could be useful in the diagnosis and prognosis of UM, due to unique miRNA profiles in melanoma cells or tissue and its association with metastasis. Although liver function tests do not provide enough data on the prognosis of UM, due to the high frequency of liver metastasis, liver function tests (LFTs) might be useful indicators; however, the absence of rising LFT values cannot lead to the exclusion of liver metastases. Molecular analysis of tumor tissue will allow us to identify patients with the added benefit of new therapeutic agents and provide a better insight into melanoma pathogenesis and its biological behavior.

## 1. Introduction

Uveal melanoma (UM) is rare, yet it is the most common primary intraocular malignancy among adults. It represents 80% of ocular melanomas and 3 to 5% of all melanoma cases [1,2]. The largest proportion of cases are derived from the choroid (90%) with other sites of tumor origin being the ciliary body (7%) and the iris (3%) [3]. UM incidence is from five to six cases per million in the United States and Europe and seven per million in Australia, respectively, which has remained unchanged over the past 30 years [1,4,5,6,7]. On the other hand, the incidence in Asia and Africa is only 0.2 to 0.3 cases per million per year [8]. Predisposing factors for UM development are age, fair skin, sensitivity to sunburn, light eye color, cutaneous, choroidal, or iris nevi. Other factors include a family history of cutaneous and uveal melanoma, dysplastic nevus syndrome, ocular or oculodermal melanocytosis—namely Nevus of Ota—and mutation of breast cancer 1 (BRCA1) associated protein 1 (BAP1) [3,6,9,10,11,12,13,14,15,16]. Risk factors for metastases also include large tumor size, epithelioid cell type, extra-scleral extension of primary tumor, chromosome 3, and loss of amplification of chromosome 8q [15,16,17].

Primary tumor treatment includes various forms and combinations of laser therapy, radiation therapy, and surgery which include local resection and enucleation of large tumors [17,18,19]. The aim of local treatment is to preserve useful vision and prevent metastatic spread; however, despite significant improvement in nearly 50% of all patients, metastatic disease will develop. When the primary tumor is diagnosed, metastases are found in less than 2% of patients, yet they can develop even over 30 years after initial diagnosis and treatment [20,21].

The 5-year survival rate of UM patients is between 50 and 70% and has remained unchanged over the past decades [2,5,9,22,23]. In the presence of metastatic disease, the median overall survival rate is 6–12 months [24,25], where 8% of patients survive for more than two years [16,17].

UM mainly spreads hematogenously, showing a tendency to metastasize in the liver (89%). Other sites include the lungs (29%) and bones (17%) [6,21,22,26]. For patients with metastatic disease, there is currently no standardized method of treatment. Potential treatment options for metastatic UM are chemotherapy, immunotherapy, and targeted therapies; however, these usually show poor outcomes [15,16,27]. Methods of treatment for patients with metastases solely in the liver include liver-directed therapies such as radiofrequency ablation, isolated hepatic perfusion (IHP), or percutan IHP [15,27]. Irrespective of the treatment options used in patients with liver metastases, the mortality rate after 2 years is nearly 90%, and approximately 1% of patients survive over 5 years. Patients with metastases in other organs or when the liver is not the primary site of metastases have a better survival rate [22,25,28]. Therefore, recognizing patients with a high risk for metastatic disease is of great value. 

In recent years, several novel treatment modalities aimed at improving tumor control and reducing unfavorable outcomes have emerged [15,21,22,26,28,29,30]. Compared to other types of melanomas, metastatic UMs have different genetic characteristics, resulting in reduced survival when traditional melanoma treatment methods are applied. Changes in the approach to treatment occurred at the beginning of 2022 when the US Food and Drug Administration (FDA) approved the use of tebentafusp for the treatment of HLA-A*02:01-positive adult patients with metastatic or inoperable UM, a bispecific glycoprotein 100 (gp100) peptide-human leukocyte antigen (HLA)-directed CD3 T-cell activator. Even though this form of treatment has not yielded the anticipated results, it does offer hope for successful targeted therapy in the near future [29,30].

More recently, the molecular, genetic, and immunobiological mechanisms involved in carcinogenesis and the progression of UM are gaining more importance [31]. A better insight into the molecular and genetic profile of UM could aid in identifying reliable prognostic and diagnostic biomarkers, modifying existing as well as developing novel therapeutic approaches specifically designed for UM patients.

## 2. Genetic Basis of Uveal Melanoma

**Figure 1:**
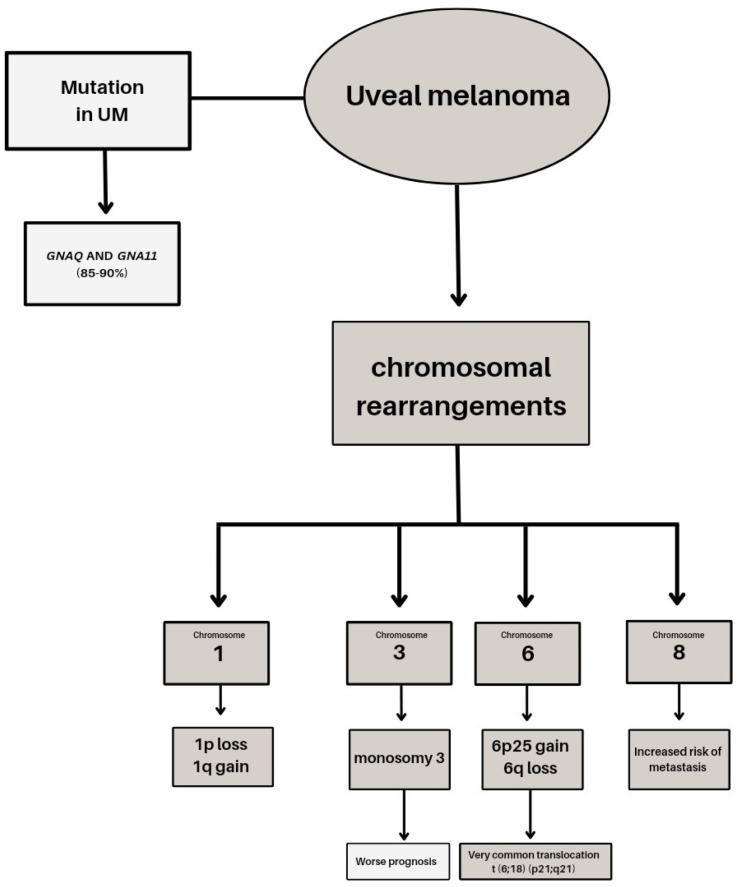
Genetic basis of uveal melanoma.

Genetic studies conducted over the years have provided valuable information about the changes observed in primary posterior uveal melanoma (UM) affecting the ciliary body and choroid. Metastases show disparate mutations than primary tumors [32]. Uveal melanomas are generally characterized by a low mutation burden and resistance to immunotherapy. UM and cutaneous melanoma are distinct genetic entities, differing not only in chromosomal changes but also in mutational signatures (Figure 1).

Cytogenetic studies have highlighted the role of genetic factors such as chromosomal aberrations and mutations in predicting patient survival. Chromosomal rearrangements in UM, particularly on chromosomes 1, 3, 6, and 8, have been extensively studied. Monosomy 3 is a frequent early event occurring in 50–60% of tumors and is often accompanied by isochromosome 8q, which leads to abnormal segregation during mitosis and high levels of 8q gain tumors with monosomy 3 or gain of 8q are associated with poor prognosis and increased risk of metastasis [33]. Other chromosomal alterations, such as gain of 6p and loss of chromosomes 1p and 8, also have significant prognostic value [34,35]. Molecular genetic analyses using DNA sequencing techniques have identified several alterations implicated in metastatic UM. Mutations in *GNAQ* and *GNA11* genes, primarily targeting codon 209, are found in up to 90% of posterior UM cases [36,37]. These mutations result in an amino acid change and are mutually exclusive. Less common mutations affecting codon 183 occur in the absence of codon 209 mutations [37]. *GNAQ* and *GNA11* genes encode the heterotrimeric Gq-proteins involved in coupling transmembrane receptors to intracellular pathways. The activation of these genes is considered an early event in UM progression [38,39]. The *BAP1* gene, located on chromosome 3, is a tumor-suppressor gene involved in cell growth and cancer pathogenesis. Inactivation or mutations of *BAP1* are present in over 80% of metastatic UMs and are associated with decreased disease-free survival rates [40]. Mutations in the *BAP1* gene lead to the premature termination of the BAP1 protein and can also impact the ubiquitin carboxyl-terminal hydrolase domain, thereby altering its deubiquitinase activity [40]. BAP1 interacts with various proteins, including the tumor suppressor gene *BRCA1*, and plays a crucial role in maintaining genome stability, regulating transcription, and responding to DNA damage [41]. Decreased disease-free survival rates are observed in tumors exhibiting monosomy 3 and *BAP1* mutations [42]. Detecting a germline mutation of *BAP1* not only indicates an increased risk of uveal melanoma (UM), but also suggests a potential association with other types of tumors. In such cases, a more comprehensive approach may be considered, involving genetic counseling and screening of family members [43]. Approximately 10–20% of uveal melanoma (UM) cases exhibit mutations in the splicing factor 3B subunit 1 (*SF3B1*) gene [44]. The *SF3B1* gene is located at chromosome 2q33 and is responsible for encoding a subunit of the spliceosome, a large complex involved in processing precursor mRNA. *SF3B1* plays a crucial role in maintaining precise splicing by retaining pre-mRNA and defining the splicing site [45]. *EIF1AX*, located on chromosome 10 encodes for the X-linked Eukaryotic Translation Initiation Factor 1A protein (Eif1A), which plays a crucial role in regulating the initiation of protein translation. Mutations in *EIF1AX* can result in the mis-selection of start sites, leading to suppressed translation of canonical transcripts or potentially upregulating oncogenes [46]. The presence of mutant *EIF1AX* has been linked to abnormal translation processes [47]. The telomerase reverse transcriptase (*TERT*) gene, located on chromosome 5p15, has been extensively implicated in various cancers [48]. However, UM exhibits a low mutation frequency of only 1% [49]. *TERT* plays a multifunctional role that encompasses the maintenance of telomere length, cell-cycle control, and DNA damage response mechanisms [50].

The *MBD4* (Methyl-CpG Binding Domain 4, DNA Glycosylase) gene encodes an enzyme that plays a crucial role in DNA repair and acts as a tumor-suppressor gene. When *MBD4* is inactivated, it results in the accumulation of a specific type of mutation, like those observed during the aging process. Germline mutations in *MBD4* are associated with a relative risk: individuals carrying this mutation have a 10 times higher risk of developing uveal melanoma compared to those without the mutation [51].

Epigenetic mechanisms play a significant role in UM, involving processes such as DNA methylation, chromatin remodeling, histone modification, and non-coding RNAs (miRNAs). Methylation events in UM can affect tumor suppressor genes, including RAS association domain family 1 isoform A (*RASSF1A*) and cyclin-dependent kinase inhibitor 2A (*p16INK4a*), which have been extensively studied in this context [52]. 

*BAP1* methylation has been recognized as a significant prognostic indicator for metastasis in UM. Robertson et al. identified four distinct UM subtypes based on the status of chromosome 3 and *BAP1* methylation: two groups associated with poor prognosis characterized by monosomy 3, and two groups with a better prognosis characterized by disomy 3. The DNA methylation pattern of *BAP1* in the first group differed from that observed in the second group [53]. In their study, Jurmeister et al. investigated the potential of DNA methylation profiling to establish a relationship between melanomas and their respective primary sites. Their findings revealed that only uveal melanomas exhibited distinct global DNA methylation profiles, displaying unique epigenetic signatures. Consequently, DNA methylation analysis allows for differentiation between uveal melanomas and melanomas originating from other primary sites [54]. 

Uveal melanomas can be categorized into prognostic groups by evaluating the expression of a specific set of genes. Initially consisting of 26 genes, this set was later refined to 15 genes. The expression pattern of these genes enables the classification of UM into low-risk (class 1) and high-risk (class 2) groups [55]. Based on the provided data set, patients with class 1 uveal melanoma (UM) exhibit a survival rate of approximately 95% at around 7 years, whereas class 2 patients have a significantly lower survival rate of approximately 30%. Further stratification within these subgroups can be achieved by considering the expression of preferentially expressed antigens in melanoma (PRAME) [56]. PRAME status has emerged as an independent prognostic biomarker for UM, specifically identifying a higher risk of metastasis in patients with Class 1 tumors. As a result, the classification of UM using genome-wide expression profiling has been revised to incorporate PRAME status. Research has indicated that when combined with a 12-gene expression panel, PRAME expression can predict a five-year metastatic rate of 0% in Class 1/PRAME− tumors, 38% in Class 1/PRAME+ tumors, and 71% in Class 2 tumors. Additionally, PRAME expression has been found to be positively associated with larger tumor diameter, and *SF3B1* mutations, as well as gains of 1q, 6p, 8q, and 9q, and losses of 6q and 11q [56]. 

There has been a notable disparity in the number of genetic studies conducted on iris melanomas compared to posterior uveal melanomas. Iris melanomas share certain genetic alterations with posterior uveal melanomas, such as deletions of 1p and alterations in chromosome 6 (both p and q). Evidence suggests that iris melanomas also exhibit abnormalities in other chromosomes, which are less commonly affected in posterior uveal melanomas. These include changes in chromosome 9p, which is more frequently associated with cutaneous melanoma, as well as rearrangements involving chromosome 18 [57]. Studies have indicated that iris melanomas exhibit mutations shared with both posterior UMs and cutaneous melanomas (CM) [58]. Specifically, mutations in genes *GNAQ/GNA11* and *BRAF* have been identified in iris melanomas [58,59] with a surprisingly high frequency of *EIF1AX* mutations [60]. An observation revealed a higher likelihood of tumor recurrence being associated with *BRAF* mutations [59]. 

Genetic studies have significantly contributed to enhancing our understanding of UM by identifying chromosomal changes and genetic mutations that can serve as valuable biomarkers for prognostic prediction. The advancements in molecular characterization of UM offer opportunities to develop targeted therapeutic strategies by focusing on relevant signaling pathways. Genetic analysis has the potential to identify patients at a high risk of metastasis, enabling more effective management and follow-up of patients.

## 3. miRNA and Uveal Melanoma

miRNAs are crucial factors that can alter the expression of genes in the regulatory mechanisms of carcinogenic processes. miRNAs are small molecules (17 - 22-nucleotides) that participate in the regulation of 60% of all protein-encoding genes and more than 400 different mRNAs [61,62]. In the development of UM, miRNAs serve as tumor suppressors or as oncogenes. Table 1 shows the literature data that includes the association between miRNAs, uveal melanoma, and their target genes. The majority of miRNAs are inhibitors of tumor development, because they participate in oncogene downregulation.

Several studies have investigated the role of miR-34a in UM. Yan et al. [63] reported that the transfection of miR-34a into UM cells leads to a decrease in cell growth and migration. This affects the downregulation of C-Met protein expression, a protein that is essential for cellular processes, and therefore indicates that miR-34a plays a role as a tumor suppressor. Furthermore, a recent study on human UM cell lines investigated the influence of miR-34a on leucine-rich repeat-containing G protein-coupled receptor 4 (LGR4), a novel target of miR-34a first discovered in retinal pigment epithelial cells. The study showed that LGR4 was upregulated in UM cells and that miRNA-34a transfection leads to reduced expression of LGR4 and causes inhibition of migration and invasion of the affected cell [64]. Previously, Dond and Lou found that two miRNAs from the same miR-34 family, miR-34b and miR-34c have also been associated with UM as suppressors of cell proliferation and migration by multiple targets. It was shown that miR-34b and miR34c inhibit cell growth and migration by blocking the cell cycle at the G1 phase and by decreasing tyrosine-kinase receptor (c-Met), cyclin-dependent kinase (CDK) 4, and CDK6 levels [65]. Downregulation of c-Met in UM cell lines was also the result of the overexpression of miR-122 and miR-144, which leads to impaired cell proliferation and migration [66]. Analyses of the miRNA in UM-cell lines and melanocytes showed significantly lower expression of miR-137 in UM cells, while its transfection into the same cells decreased cell growth and affected the downregulation of CDK6 and oncogene microphthalmia-associated transcription factor (*MITF*) [67]. *MITF* was also downregulated by miR-182 [68].

Additionally, miR-124a also acts as a tumor suppressor of UM, as it inhibits cell growth, migration, and invasion by affecting multiple targets such as CDK4, CDK6, cyclin D2 and enhancer of zeste homolog 2 (EZH2) [69]. It is known that CDK4 and cyclin D2 are essential for cell cycle G1 phase [70,71], while EZH2 is a protein that serves as a histone lysine methyltransferase and was previously found to be associated with the progression of skin melanoma [72]. Li et al. in a recent study reported lower expression of miR-145 and miR-205 in clinical samples taken from patients with UM than in normal tissue samples [73]. Furthermore, overexpression of those two miRNAs decreases the level of cell division control protein 42 (CDC42), which is associated with many cancers in humans, such as cervical squamous cell carcinoma, cutaneous melanoma, sarcoma, uterine carcinosarcoma, and others [74]. The list of miRNAs that act as tumor suppressors in UM additionally includes miR-9 [75], miR-17-3p [76], miR-142-3p [77], miR-216a-5p [78], and miR-224-5p [79]. miR-216a-5p inhibits hexokinase-2 (HK2) expression and therefore reduces glycolysis, glucose uptake, ATP production, extracellular acidification rate (ECAR), and an increase in oxygen consumption rate (OCR) [78].

In addition to acting as tumor suppressors, miRNAs in UM can also play the role of oncogenes and therefore promote disease progression and initiation. It was noted in the literature that miRNA-21, which could be a potential biomarker as well as a therapeutic target in colon cancer, was the most investigated [80]. Overexpression of miR-21 in UM cell lines promotes cell proliferation, inhibits the expression of the *p53* gene, and increases LIM and SH3 protein 1 (LASP1) [81].

A study conducted on tumor samples obtained from patients with choroidal UM reported upregulation of miR-155 in both melanoma cells and tissues [82]. Additionally, transfection of miR-155 into the cells causes an increase in cell growth and inhibits the Nedd4-family interacting protein 1 (NDFIP1). This suggests that miR-155 might serve as a potential therapeutic target in UM [82]. Cell cycle progression in UM was also induced by upregulation of miR-181, especially miR-181b which inhibits expression of CTD small phosphatase-like tumor suppressor (*CTDSPL*), an activator of the downstream effector E2F, which leads to the disruption of cell cycle control [83]. Previous studies have reported that expression of miR-367 and miR-454 decreases levels of phosphatase and the tensin homolog (*PTEN*) [84,85]. *PTEN* is involved in the phosphatidylinositol-3-kinase (PI3K) and serine/threonine-protein kinase (AKT) pathways. Low expression of *PTEN* is associated with the progression of disease and metastasis formation [86]. A recent study reported higher expression of miR-652 in UM cell lines in comparison to melanocyte cells and non-tumor tissues [87]. The target gene of miR-652 is oncogene homeobox A9 (*HOXA9*), and downregulation of miR-652 increased HOXA9 expression in cells [87].

A study conducted by Souri et al. compared the expressions of HLA and miRNA in the same tumors using mRNA expression with four unlined HLA Class I probes [88]. The study showed that one cluster of miRNAs was positively matched with HLA Class I and infiltrating leukocytes, while the other showed the opposite effect. The study also confirmed the relationship between both miRNA expression patterns and chromosome 3 (BAP1) status in UM. This finding could be the starting point to consider miRNAs as regulators of inflammation in UM, regulated by BAP1 [88].

**Table 1:** The role of miRNAs found to be associated with uveal melanoma (UM) in humans.

miRNAs	Sample Type	Role	Regulation Action	Target	Reference
miR-9	cells with highly and poorly invasive potential	tumor suppressor	suppresses cell migration and invasion	downregulation NF-κB1	[75]
miR-17-3p	UM patients tissues and cell lines	tumor suppressor	inhibiting cell proliferation, migration and invasion	decreased MDM2 expression	[76]
miR-20a	cells and melanocytes, and tumor tissue samples for patients	oncogenic	increases cell growth, migration and invasion activities	not validated	[89]
miR-21	cell lines and tissue samples	oncogenic	promoted proliferation, migration, and invasion	inhibited expression p53	[81]
miR-34a	cells lines and melanocytes	tumor suppressor	inhibiting cell proliferation, migration and invasion	downregulation c-Met; decreased LGR4	[63,64]
miR-34b/c	cells and melanocytes from patients, the uveal stromal tissues	tumor suppressor	reduction in cell growth and migration	downregulation c-Met	[65]
miR-122	cell lines and patients’ tissues	tumor suppressor	impaired cell proliferation and migration	reduced expression ADAM10 and c-Met	[66]
miR-124a	cell culture and tumor specimens	tumor suppressor	inhibited cell growth, migration and invasion	downregulation CDK4, CDK6, cyclin D2 and EZH2	[69]
miR-137	cells lines from patients	tumor suppressor	decrease cell growth	downregulated MITF and CDK6	[67]
miR-142-3p	cells and uveal melanocytes from patients	tumor suppressor	inhibited cell proliferation, migration and invasiveness	reduced CDC25C, TGFβR1, GNAQ, WASL, and RAC1	[77]
miR-144	cell lines and patients tissues	tumor suppressor	impaired cell proliferation and migration	reduced expression ADAM10 and c-Met	[66]
miR-145	cells with highly and low invasive potential and patients’ tissues	tumor suppressor	reduce proliferation, migration and invasion	downregulation CDC42	[73]
miR-155	cell culture and tumor specimens	oncogenic	increase cell growth and invasion	inhibited NDFIP1	[82]
miR-181	cell lines	oncogenic	promoted cell cycle progression	inhibited CTDSPL	[83]
miR-182	cells lines from patients	tumor suppressor	decrease cell growth, migration, and invasiveness	downregulation MITF, BCL2 and cyclin D2	[68]
miR-205	cells with highly and low invasive potential and patients’ tissues	tumor suppressor	reduce proliferation, migration and invasion	downregulation CDC42	[73]
miR-216a-5p	cell lines and human embryonic kidney cell line	tumor suppressor	reduce cell proliferation	inhibit HK2 expression	[78]
miR-222	cell lines	oncogenic	increases proliferation and migration	decreased PI3K, Akt, MMP-9	[87]
miR-224-5p	cell lines and human embryonic kidney cell line and patients’ tissues	tumor suppressor	inhibited capacities of proliferation, invasion and migration	decreased PIK3R3 and AKT3	[79]
miR-296-3-p	choroidal tissues	tumor suppressor	inhibiting cell proliferation, cell cycle progression, migration	targeting of MMP-2 and MMP-9 in combination with FOXCUT	[90]
miR-367	tissue specimens, cell culture	oncogenic	promote cell proliferation and migration	reduced PTEN	[84]
miR-454	tumor samples from patients	oncogenic	promote cell proliferation, colony formation, invasion and induction	reduced PTEN	[85]
miR-652	cell lines and tissues samples from patients	oncogenic	increases proliferation and migration	decrease HOXA9	[91]

NF-κB1: nuclear factor kappa B cells 1; MDM2: murine double minute clone 2 oncoprotein; LGR4: leucine-rich repeat-containing G protein-coupled receptor 4; ADAM10: disintegrin metalloproteinase 10; c-Met: tyrosine-kinase receptor; CDK4: cyclin dependent kinase 4; CDK6: cyclin dependent kinase 6; cyclin D2: protein coding gene; EZH2: enhancer of zeste homolog 2; MITF: microphthalmia associated transcription factor; CDC25C: cell division cycle 25 homolog c; TGFβR1: transforming growth factor beta receptor 1; GNAQ: guanine nucleotide-binding protein alpha-Q; WASL: Wiskott–Aldrich syndrome like; RAC1: ras-related c3 botulinum toxin substrate 1; CDC42: cell division control protein 42; HK2: hexokinase-2; PI3K: phosphatidylinositol-3-kinase; AKT: serine/threonine-protein kinases; PIK3R3: subunit of phosphatidylinositol 3-kinase; p53: tumor protein; NDFIP1: nedd4-family interacting protein 1; BCL2: B-cell lymphoma 2; MMP-9: matrix metallopeptidase-9; PTEN: phosphatase and tensin homolog; HOXA9: Homeobox A9; FOXCUT: FOXC1 promoter upstream transcript.

### miRNAs as Biomarkers

A study conducted on fresh frozen UM tissue reported the association between miRNA expression and melanoma metastasis [92]. The authors used a multiplexed microarray-based platform to identify miRNA profiles in two classes of tumor tissues, low (class 1) and high metastatic risk (class 2). They found six miRNAs (let7b, miR-miR-199a-3p/5p, miR-143, miR-193b, and miR-652) to be upregulated in high metastatic risk tissue samples, while two of them, let-7b and miR-199a, had the strongest association, which makes them more accurate predictors of metastasis [92].

**Figure 2:**
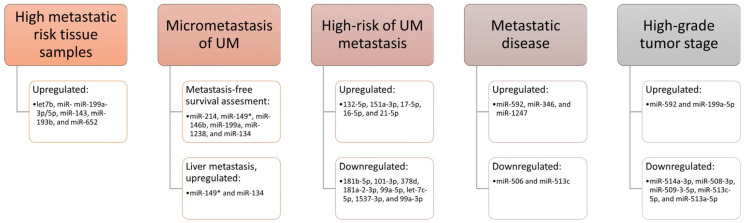
miRNA as a risk assessment for the development of metastases (studies with different designs) [92,93,94,95,96].

Venkatesan and co-authors [93] studied the involvement of miRNAs in the micrometastasis of UM. They found that miR-214, miR-149*, miR-146b, miR-199a, miR-1238, and miR-134 may be used to assess metastasis-free survival in patients with UM, while miR-149* and miR-134 have a significant association with liver metastasis. A recent study used a next-generation sequencing approach and identified thirteen miRNAs with expressions that were different in the high-risk group of UM in comparison to the low- and intermediate-risk groups [94]. Five of them (132-5p, 151a-3p, 17-5p, 16-5p, and 21-5p) were upregulated, while eight (181b-5p, 101-3p, 378d, 181a-2-3p, 99a-5p, let-7c-5p, 1537-3p, and 99a-3p) were downregulated in the group at high risk for UM-metastasis, indicating that some miRNAs have a potential role in future treatment. Furthermore, studies conducted on patients with primary or metastatic UM tumors reported increased expression of miR-592, miR-346, and miR-1247, and decreased expression of miR-506 and miR-513c in patients with metastatic disease [95]. These findings showed that selected miRNAs may serve as early biomarkers of disease progression. miRNAs as a risk assessment for the development of metastases are summarized in Figure 2.

In addition, some authors used the data on miRNA expression contained in the public database ‘The Cancer Genome Atlas’ (TCGA) to identify miRNAs that can be used as biomarkers in clinical practice. They found that miR-514a-3p, miR-508-3p, miR-509-3-5p, miR-513c-5p, and miR-513a-5p were downregulated, while miR-592 and miR-199a-5p were upregulated in the high-grade tumor stage compared to the low-grade tumor stage and in the deceased group vs. the alive group regarding vital status [96]. On the other hand, Xin and co-authors used a linear prognostic model of nine miRNAs (miR-195, miR-224, miR-365a, miR-365b, miR-452, miR-4709, miR-7702, miR-513c, and miR-873) to divide patients with UM into a high- and a low-risk group [97]. The overall survival was shorter in the high-risk group than in the low-risk group. They identified 418 genes as target genes of these 9 miRNAs, which were involved in the activity of protein binding and phosphorylation and the transforming growth factor beta (TGF-β) pathway [97]. 

The association of miRNA expression, metastasis, and survival in patients with UM using the TCGA dataset was also investigated by Vashishtha et al. [98]. They reported 22 miRNAs, 3 upregulated (miR-199a-5p, miR-708-5p, and miR-592), and 9 downregulated (miR-508-3p, miR-509-3p, miR-508-5p, miR-514a-3p, miR-506-3p, miR-509-3-5p, miR-513c-5p, miR-513a-5p, and miR-513b-5p) in patients with vs. those without metastatic UM. The overall survival in patients with UM was associated with 15 miRNAs, 11 miRNAs with a hazard ratio <0.10, and 4 miRNAs with a hazard ratio >10. Furthermore, tumor specimens obtained from formalin-fixed paraffin-embedded tissue of 52 cutaneous melanomas and 41 UMs had lower expression of miR-15a, miR-185, and miR-221 and higher levels of IL-10Rα than normal skin specimens [99]. Expression of IL-10Rα in melanoma cells is regulated by miR-15a, miR-185, and miR-211, and the knockdown of these miRNAs supported proliferation in the cell lines.

Circulating miRNAs were investigated as diagnostic and prognostic biomarkers due to their stability in the serum, high specificity and selectivity, and non-invasive sampling method. Triozzi et al. [100] investigated miRNA expression in the plasma of UM patients during 33 weeks of therapy with dacarbazine and interferon-alfa-2b, and in follow-up patients after 6 months from their last therapy. They observed downregulation of miR-126 and miR-199a and upregulation of miR-16 and miR-106a after therapy only with interferon-alfa-2b. The limitation of this study was the lack of risk data for metastasis during follow-up. The same group of authors also investigated miRNA profiles in the plasma of 10 patients with monosomy-3 and 10 without it [101]. They found that 15 miRNAs had different expression between patients with monosomy-3 and controls, 11 miRNAs (miR-191, miR-93, miR-221, miR-342-3p, miR-19b, miR-199a-5p, miR-25, miR-27a, miR-23a, miR-15b, and miR-223) were upregulated, and 4 (miR-1227, miR-663, miR-654-5p, and miR-1238) were downregulated. Furthermore, in this study, they investigated differences in miRNA expression in patients with and without metastatic disease during follow-up. Metastatic patients had seven downregulated miRNAs (miR-509-3-5p, miR-508-3p, miR-506, miR-513a-5p, miR-509-3p, miR-513b, miR-935) and one upregulated miRNA (miR-624) when compared to non-metastatic patients. Stark et al. analyzed [102] miRNA expression in the serum of participants with uveal nevi and patients with localized and metastatic UM. The expression of six miRNAs (miR-16, miR-145, miR-146a, miR-204, miR-211, and miR-363-3p) was significantly different among those three groups, whereas miR-211 was shown to be a discriminator between metastatic disease and localized UM. Downregulation of six miRNAs (miR-19a, miR-30d, miR-127, miR-451, miR-518f, miR-1274b) and upregulation of two miRNAs (miRNA-146a, miR-523) were found in the serum of patients with UM vs. controls [103]. After singular validation of miR-146a and miR-523 as potential markers for disease, only miR-146a was overexpressed in patients’ serum and UM cells compared to controls. On the other hand, patients with UM had higher expression of miR-20a, 125b, 146a, 155, 181a, and 223 in plasma samples than controls. Also, these miRNAs increased with metastasis, with the exception of miR-181a, which decreased.

In summary, for a better understanding of the role in UM, using miRNAs as predictive markers or potential biomarkers requires further studies on a larger number of samples/patients, adequate bioinformatics tools for predictive analysis, clinical validation of findings such as the comparison of early and late metastasis. However, miRNAs have a future in the diagnosis and prognosis of UM, due to unique miRNA profiles in melanoma cells or tissue and their association with metastasis.

## 4. Uveal Melanoma and DNA Methylation

DNA methylation, one of the most prevalent epigenetic forms, controls the expression of genes in two different ways: either by inhibiting the activity of transcriptional proteins, which prevents their binding to the gene, or by methylated DNA binding methyl-CpG-binding domain proteins, resulting in a buildup of inactive chromatin [104].

According to a study by Bakhoum, the levels of BAP1 protein, *BAP1* mutations, and *BAP1* genomic copy loss are all correlated with the methylation of BAP1 at a single genomic locus. They concluded that this offers helpful prognostic information, even in tumors where whole-exome sequencing failed to identify any *BAP1* mutations, and that UM metastasis is associated with BAP1 deletion in the primary tumor [105]. In the bone, skin, and liver, UM metastasis was found to be caused by aberrant methylation of *BAP1* and *SF3B1*. The study demonstrated a significant degree of patient variability and that epigenetic alterations in metastasized UM lead to the altering of many tumor-related genes [106]. Genes linked to early metastasis and a poor prognosis were discovered using integrated differential DNA methylation and gene expression study samples. While *TMEM200C*, *RGS10*, *ADAM12*, and *PAM* are hypomethylated and candidate oncogenes connected to early metastasis, *RNF13*, *ZNF217*, and *HYAL1* are hypermethylated and candidate tumor suppressors [107]. As a result of their down-regulated expression, the tumor suppressor genes encoding *p16INK4a* (cyclin-dependent kinase inhibitor 2A), *RASSF1A* (RAS association domain family 1 isoform A), and *p16INK4b* (RAS association domain family 1 isoform B) may play a significant role in tumorigenesis. The tumor suppressor gene is less frequently hypomethylated than it is hypermethylated. However, it was discovered that the *DSS1* gene and preferentially expressed antigen in melanoma (PRAME), which is a reliable indicator of metastasis in uveal melanoma, are overexpressed in UM as a result of hypomethylation [52].

A recent study using UM tissue from 68 UM patients in Slovakia revealed that high-risk tumors had a total of 7810 CpGs that were hypomethylated and 16,588 that were hypermethylated. Three genes with hypomethylation or upregulation, *HTR2B* (FC 191.4), *AHNAK2* (FC 12.6), and *CALHM2* (FC 7.8), as well as six genes with hypermethylation or downregulation, *SLC25A38* (FC-4.6), *EDNRB* (FC-4.7), *TLR1* (FC-8.6), *RNF43* (FC-10.8), *IL12RB2* (FC-18.1), and *MEGF10* (FC-25) were selected for validation and the number of CpGs inversely correlated with gene expression [108]. 

All of the studies mentioned above demonstrated the important part that DNA methylation has in UM development, but more research is needed to determine the diagnostic utility and potential future applications of this sort of epigenetic analysis.

## 5. Uveal Melanoma Biomarkers in Body Fluids

Many studies have implicated the interpretation of different serum biomarkers in uveal melanoma. This included the determination of liver function tests (LFT), tumor markers, growth factors, and other proteins. The results showed high levels of heterogeneity.

### 5.1. Liver Function Tests as Uveal Melanoma Biomarkers

Due to very frequent metastases in the liver, LFT measurements are routine diagnostics during check-ups of patients with UM. The diagnostic and prognostic value of LFTs showed some contradictions among different studies. A study conducted on 88 Canadian patients from Quebec who developed metastases detected during a semiannual follow-up showed that overall LFT sensitivity ranged between 12.5% and 58.0%, while the positive predictive value (PPV) ranged between 9.4% and 38.6%. This implies that LFT might be useless in the detection of early metastasis [109]. A total of 95 Finnish patients with systemic metastases participated in an annual review that included LFTs (AST, ALT, AP, and LDH), a chest radiogram, and liver imaging. The results showed that AP in serum was higher than the upper reference limit in 30%, LDH in 63%, AST in 36%, and ALT in 36% of patients [110]. A US study performed on 505 medical records of patients with UM selected 76 patients with liver metastasis according to a study protocol. In 69% of patients, at least one of the LFTs was increased: 55% showed an increased value of AST, ALT, or both, 43% AP, and 33% had an increased concentration of bilirubin [111]. A prospective study on 102 French patients with UM did not show sufficient accuracy of LFT screening in the detection of liver metastases [112].

We can conclude that liver function tests do not provide enough data for the prognosis of uveal melanoma. Indeed, due to the high frequency of liver metastases, LFTs might be useful indicators, but the absence of rising LFT values cannot lead to the exclusion of liver metastases. Therefore, all alternative and more selective and accurate diagnostic methods must be performed during a patient’s follow-up.

### 5.2. UM-Serum Biomarkers

Investigation of serum tumor markers in UM mostly reflects their capacity to improve the prognosis of uveal UM metastases. S-100β, melanoma-inhibitory activity (MIA), and osteopontin (OPN) serum concentrations, as well as concentrations of some growth factors and cytokines, might serve as important prognostic tools in UM.

A study conducted on serum samples from the Ocular Oncology Serum Bank at Hadassah University Hospital showed that the concentrations of osteopontin OPN, MIA, and S-100β significantly correlate with liver metastases of UM. The combination of these markers proved to be very sensitive and indicative of the detection of liver metastases [113]. The same group of authors conducted a study that included 43 patients without UM disease (DF) for at least 10 years, 32 patients with metastatic UM, and 53 healthy subjects. Their findings show that concentrations of the tumor markers OPN, S-100β, MIA, and tissue-specific polypeptide antigen (TPS) increased before confirmation of metastases by imaging techniques [114]. This indicates that routine measurements of these biomarkers in serum could be important for the early detection of liver metastases and an early therapeutic approach. OPN and MIA were found to be significant tumor markers in German patients with metastatic UM, as both markers were markedly higher in patients with metastases than in those without metastases [115]. A case-control study performed on patients from the Netherlands showed that mean serum concentrations of S-100B and MIA were significantly higher in patients with metastases compared to melanoma patients without metastases [116]. Early detection of tumor progression was also studied in serum exosomes as a carrier of different tumor progression biomarkers. A study involving 20 Polish patients with primary or metastatic UM and 20 healthy donors showed that the concentrations of several molecules associated with inflammation, such as interferon-gamma, IL-2, IL-22, IL-12, Pentraxin-3, TNFSF13B, and TNFSF8, were significantly higher in exosomes from subjects with metastatic UM than in exosomes from healthy donors [117]. Serum concentrations of IGF-1 in patients from Israel indicated that this biomarker could be useful for predicting UM metastases, as IGF-1 was significantly lower in subjects with metastatic tumors compared to subjects who recovered from the disease 10 or more years ago (or even more) than in control healthy subjects [118]. A study of 45 patients with immune-related adverse events (irAEs) in patients with metastatic UM treated with an immune checkpoint inhibitor (ICI) showed that serum CRP and IL-6 concentrations may be useful markers for the early detection of irAEs [119].

Through a comparison of studies that investigate LFTs and some tumor biomarkers in serum, we can conclude that LFTs have an indicative impact on the prognosis of metastatic events in UM patients. Furthermore, additional biomarkers with higher sensitivity must be determined during patient follow-ups. OPN, MIA, and S-100β seem to be the markers of choice, but some other serum biomarkers like them need to be further investigated because the etiology of UM is still unclear. This also includes prognostic biomarkers that can be used in the diagnosis and prognosis of disease progression.

### 5.3. Ocular Fluid Proteins as Biomarkers in Uveal Melanoma

*Aqueous humor* (liquid fluid) and *vitreous humor* (gel-like fluid) are fluids localized in front of and behind the lens, respectively [120]. The composition of *aqueous humor* includes organic and inorganic ions, glutathione, carbohydrates, amino acids, carbon dioxide, oxygen, and water [121]. Depending on the various eye disorders, the testing of several types of cytokines, chemokines, growth factors, and other proteins can play an important role in the diagnosis of various eye diseases. Those proteins might serve as biomarkers in the diagnosis, prognosis, and treatment of uveal melanoma (UM). Several studies investigated the difference in protein composition of the *aqueous humor* between patients who suffered from uveal melanoma and subjects with different benign ocular disorders.

Expression of many angiogenic, chemotactic, and inflammatory cytokines was significantly different between Chinese patients with UM and control subjects with cataracts. This included interleukins IL-6 and IL-8, interferon-inducible protein-10 (IP-10), placental growth factor-1 (PLGF-1), monocyte chemoattractant protein-1 (MCP-1), nerve growth factor-b (NGF-β), epidermal GF (EGF), b-fibroblast GF (b-FGF), vascular endothelial GF-A (VEGF-A), and regulated upon activation, normal T cell expression, and secreted chemokine (RANTES) [122]. Similar studies were performed on 35 Italian subjects with UM and 35 controls with cataracts. The results showed higher concentrations of IL-6, IL-8, EGF, bFGF, macrophage inhibitory factor (MIF), and MCP-1 in the *aqueous humor* of patients with UM compared to subjects with cataracts [123].

The protein composition of patients with UM was also investigated in *vitreous humor*. Comparison of Dutch patients with UM and controls revealed results that demonstrated higher *vitreous* fluid concentrations of IL-6, IL-8, IP-10, MCP-1, macrophage inflammatory protein 1α (MIP-1α), MIP-1β, TNF-α, and RANTES. It was also shown that IL-6, IL-8, IP-10, MCP-1, MIP-1α, MIP-1β, TNF-α, RANTES, GCSF, IFN-γ, and VEGF concentrations positively correlate with tumor size [124]. A comparison of *aqueous humor* composition between Japanese subjects with UM and some benign eye tumors showed significantly higher concentrations of IL-8, MCP-1, and angiogenin. These findings indicate that IL-8, MCP-1, and angiogenin might serve as potential biomarkers for differentiation between malign UM and benign intraocular tumors [125].

The measurements of biomarkers in *vitreous* and *aqueous humor* of Austrian patients with UM showed higher concentrations of Flt-3 ligand, IL-6, IL-8, (IP)-10, MCP-1, MIP-1α, platelet-derived growth factor AA (PDGF-AA), and VEGF when compared with subjects with a cataract [126]. Eotaxin was significantly higher in the *aqueous*, and IL-7 in the *vitreous humor* of patients with UM. Tumor dimensions showed positive correlations with IP-10, and MIP-1 (in *aqueous* and *vitreous humor*)*,* while Fms-related tyrosine kinase 3 ligand (FLT3LG), IL-6, IL-8, and MCP-1 were positively correlated in *vitreous humor*. Tumor infiltration in Bruch’s membrane, an extracellular matrix between the retinal pigment epithelium and the choroid, showed a positive correlation with FLT3LG and MCP-1 (in *aqueous* and *vitreous humor*), and IL-8, IP-10, MIP-1α, and PDGF-AA (in *vitreous humor*) [126].

Soluble human leukocyte antigens (sHLA) were investigated in Dutch patients with UM. In patients with sHLA-positive *aqueous humor*, the frequency of metastases was higher, and they showed significantly worse survival [127].

Overall, we can conclude that different case-control studies involving UM and benign eye disorders have revealed the role of angiogenic, inflammatory, and chemotaxic biomarkers as significant factors supporting the association between inflammation and tumorigenesis.

In addition to this diagnostic feature of cytokines and growth factors, biomedical research results in the measurement of biomarkers that can be useful for determining treatment effect and prognostic value. 

A study involving 18 Chinese patients who underwent iodine-125 plaque therapy showed a significantly strong positive correlation of *aqueous humor* VEGF-A and PLGF levels with tumor thickness, and the correlation remained the same after radiation therapy [128]. Additionally, the expression of both growth factors increased after the surgery. IFN-γ, IL-2, and IL-10 concentrations were also observed to be significantly different between subgroups with elevated and non-elevated VEGF-A and PLGF, suggesting their potential role in inflammation and angiogenesis after radiotherapy [128]. The effect of combined Ru-106 brachytherapy and transpupillary thermotherapy (TTT) was investigated in 20 South Korean patients. An increase in the expression of IL-6, IL-8, and IL-1β was observed. This could serve as a useful tool for detecting radiation-related side effects [129]. Analysis of *aqueous humor* from 83 Dutch patients who underwent primary enucleation for UM showed a positive association between high ANG-2 expression, the development of metastases, and the presence of monosomy-3 [130]. Therefore, ANG-2 may be useful as an effective target for the potential treatment of UM.

The concentrations of different cystatins were investigated in different body fluids of Russian patients with UM, intraocular fluid, tears, and sera, and the results showed the diagnostic potential of cystatins [131]. Cystatins, as reversible inhibitors of cysteine proteases, have an important role in tumor progression.

The most frequently investigated biomarkers and their significance in the diagnosis, prognosis, and treatment of UM are listed in Table 2.

## 6. Current Challenges and Clinical Implications

UM is a serious and rare disease and irrespective of the modality of treatment administered over 50% of patients will develop fatal metastasis within ten years after initial diagnosis, most commonly in the liver [132]. Given that the occurrence of systemic disease is associated with increased mortality and adverse outcomes, it is important to identify patients at high risk. Once metastases develop, life expectancy is reduced, as existing treatment methods are still relatively ineffective [16,25,133].

### 6.1. Metastatic Risk Assessment

To date, treatment of metastatic UM has not given satisfactory results and as such remains a challenging problem for specialists dealing with ocular oncology [9,15,17,22,23,25,26,27,28,29,30,134]. In spite of long-term research, the only established method for controlling the spread of the disease is early treatment aimed at removing liver metastases, which has provided benefits for some patients [15]. Therefore, a better insight into molecular, genetic, and immunobiological features, early detection, and new effective treatment strategies are essential. Research in this area may aid in identifying valuable biomarkers for diagnostic and prognostic purposes and new treatment methods with the aim of selecting patients who would achieve the most benefit. Assessment of the risk for metastases could be achieved by genetic and epigenetic profiling of UM tumor tissue [21,24,31,135,136,137]. Future research should take into consideration how the tumor cells evade the immune system in order to develop new therapies that would affect these pathways. 

Understanding the processes involved in drug resistance and side effects could also significantly improve treatment outcomes in patients with metastatic tumors and setting guidelines for novel treatment options [15,31,138,139,140]. Research should therefore be aimed towards applying the available knowledge regarding molecular, genetic, and immunobiological features of UM and directing them into effective forms of treatment targeting UM-specific molecular variations.

### 6.2. Tumor Tissue Analysis

Biopsy of primary UM is a valuable method that provides us with data pertaining to the morphology of tumor tissue, its immunological, cytological, biochemical, histopathological, and genetic features. The analysis of the samples obtained at the time of diagnosis and treatment of the primary tumor is essential for the assessment of its malignant and metastatic potential as well as the selection of the most effective treatment approach [3,9,21,25,31,55,132,133,138,139]. Fine-needle aspiration biopsy (FNAB) is the most commonly used method for biopsy of the primary UM, while other methods include incisional biopsy, with or without vitrectomy, and excisional biopsy. These are invasive techniques with a possible risk of tumor spread eye complications or insufficient sampling. However, if performed by an experienced eye surgeon, biopsy is a safe procedure whose benefits achieved by the progress in diagnostic and prognostic analysis of UM specimens outweighs the potential risks [3,9,135].

A new approach that uses liquid biopsies to analyze blood and other body fluids, such as *vitreous* and *aqueous humor* samples, may represent an additional less invasive method of analysis. In the future, it could be useful in diagnosis, monitoring tumor progression, determination of diagnostic and prognostic biomarkers, early detection of metastatic disease, local tumor recurrence, and response to treatment [21,135,137]. Nevertheless, histopathological, cytological, biochemical, and genetic analyses of tumor samples obtained by biopsy are still necessary to assess the malignant and metastatic potential of UMs [3,21,135]. Current evidence suggests that liquid biopsies may be a source of biomarkers, but in the absence of standardized methodology and analysis, further research is needed to justify the use of liquid biopsy methods in the diagnosis and follow-up of patients with UM [21,135,141].

Genetic and epigenetic features of tumors have attracted increasing attention in recent years due to their significant role in the process of carcinogenesis and the possibility of a better understanding of tumor behavior. This could enable the development of reliable biomarkers and a new therapeutic approach, resulting in new discoveries in the treatment of UM, better survival, and giving hope to patients who develop metastases [31,32,139].

### 6.3. Novel Treatment Options for Metastatic Uveal Melanoma

The treatment options for metastatic UM, albeit with some limitations, have improved with the use of tebentafusp, an immunotherapeutic agent consisting of an engineered T-cell receptor aimed at the gp100 epitope presented by HLA A*02:01 cells, fused to an anti-CD3 single-chain variable fragment. However, its action is limited to patients who have an HLA-A*02:01 allele with an additional drawback being the need for intensive monitoring and a weekly dosing schedule [142]. Further progress in the treatment of metastatic UM is represented by studies on the combined use of tebentafusp together with immune checkpoint inhibitors. Future trials could evaluate its role in the adjuvant therapy for patients with high-risk UM and molecular, genetic, and immunobiological analysis could give a significant contribution in this area [29,30,142].

## 7. Conclusions

UM is a life-threatening and relatively rare cancer that exhibits markedly distinctive biological behavior when compared to other types of melanomas and therefore necessitates particular treatment approaches. Despite its rarity, it presents a problem since the outcome continues to be unfavorable in a significant proportion of cases. To date, regardless of advancements in the diagnosis and treatment of primary tumors, metastatic disease continues to pose a challenge due to the lack of available successful therapeutic treatment options.

Molecular research has given a better understanding of the genetic and epigenetic mechanisms involved in the biological behavior of UM. In order to improve the treatment of these patients, emphasis needs to be aimed on researching diagnostic, prognostic, and therapeutic biomarkers, tumor immunogenicity, and the underlying mechanisms of tumor formation and spread. Better knowledge regarding the molecular changes underlying the development of UM promises a new perspective for improved and targeted therapeutic agents that influence certain phases of tumor development with a personalized approach. This will undoubtedly lead to advances in the systemic treatment of patients with metastatic disease as well as the prevention of spread in those having tumors with high metastatic potential. Molecular analysis of tumor tissue specimens will allow us to identify patients who would benefit from new therapeutic agents and provide a better insight into melanoma pathogenesis and its biological behavior.

## Figures and Tables

**Table 2:** The roles of different body fluid biomarkers in diagnosis prognosis and treatment of uveal melanoma.

Sample Type	Biomarker(s)	Type of Molecules	Significancve in Diagnosis and Prognosis	References
aqueous humor	IL-6, IL-7, IL-8, IP-10, PGF-1, MCP-1, NGF-β, EGF, b-FGF, PDGF-AA, VEGF-A, RANTES, MIP-1α, MIP-1β, TNF-α, eotaxin	cytokines, growth factors	significantly higher in UM than cataract	[122,123,124,126]
aqueous humor	IL-8, MCP-1, angiogenin	cytokines, growth factors	differentiate between UM and benign ocular tumors	[125]
vitreous humor	Flt-3 ligand, IL-6, IL7, IL-8, IP-10, MCP-1, MIP-1α, PDGF-AA, VEGF	cytokines, growth factors	significantly higher in UM than cataract	[126]
aqueous humor	IP-10, MIP-1α	cytokines, growth factors	positive correlation with UM-tumor dimensions	[126]
vitreus humor	IP-10, MIP-1α, FLT3LG, IL-6, IL-8, MCP-1	cytokines, growth factors	positive Correlation with UM-tumor dimensions	[126]
aqueous humor	sHLA	antigen	metastases, worse survival	[127]
aqueous humor	VEGF-A and PLGF	growth factors	positive correlation with tumor thickness and increaly expressed after the surgery	[128]
aqueous humor	IL-6, IL-8 and IL-1β	growth factors	increased expression after Ru-106 brachytherapy and transpupillary thermotherapy	[129]
aqueous humor	ANG-2	angiognesis	metastasis	[130]
tears	Cystatin C	proteases inhibitor	diagnosis of UM	[131]
serum	LFT	enzymes and bile pigments	metastasis, but with low predictive value	[109,110,111,112]
serum	OPN, MIA and S-100β	tumor markers	UM hepatic metastases	[113,114,115,116]
exosomes	interferon-γ, IL-2, IL-22 and IL-12, Pentraxin-3, TNFSF13B and TNFSF8	interleukins and other inflammatory-related molecules	UM metastases	[117]
serum	IGF-1	growth factor	prediction of metastases	[118]
serum	IL-6, CRP	interleukins and inflammatory-related molecules	early detection of irAR	[119]

ANG-2: angiogenin; 2b-FGF: b-fibroblast; CRP: C-reactive protein; EGF: epidermal growth factor; FLT3LG: Fms-related tyrosine kinase 3 ligand; IP-10: interferon inducible protein-10; IL: interleukin; LFT: liver function tests; MIP-1α & MIP-1β: macrophage inflammatory protein 1α & 1β; MCP-1: monocyte chemoattractant protein-1; MIF: macrophage inhibiting factor; NGF-β: nerve GF-β; PLGF-1: placental growth factor1; OPN: osteopontin; PDGF-AA: platelet-derived growth factor AA; RANTES: regulated upon activation; sHLA: soluble human leukocyte antigens; TNF-α: tumor necrosis factor α; TNFSF13B & 8: TNF receptor superfamily member 13B & 8; VEGF-A: vascular endothelial GF-A.

## Data Availability

Not applicable.
